# Autoimmune Encephalitis Presenting as Dystonia-Parkinsonism

**DOI:** 10.31486/toj.21.0131

**Published:** 2022

**Authors:** Sophia E. Milburn, Colin Van Hook, Andrew J. Steven

**Affiliations:** ^1^ Department of Radiology, Ochsner Clinic Foundation, New Orleans, LA; ^2^ Department of Neurology, Ochsner Clinic Foundation, New Orleans, LA

## INTRODUCTION

Autoimmune encephalitis refers to a variety of conditions in which the body's immune system attacks the brain. The disease may present acutely but can also persist through a chronic progressive or relapsing-remitting time course.^1^ The pathology of autoimmune encephalitis is either antibody- or T-cell–mediated inflammation of the brain.^1^ Some cases of autoimmune encephalitis are the result of an immune response to tumors outside of the central nervous system, referred to as paraneoplastic.^1^ This often-elusive diagnosis can be suspected based on patient history and certain characteristic imaging findings, with the diagnosis confirmed by the identification of specific serologic markers.

## CASE DESCRIPTION

A nondiabetic male developed subacute onset of back pain, speech changes, and balance difficulty at age 47 years. On initial examination, he was noted to have dystonic speech, coarse resting and postural tremor, and increased tone with mild bradykinesia and dysmetria in the right arm. He had no abnormalities in tone or abnormal movements of the left arm. Tone was subjectively increased in the bilateral low extremities, with more severe bradykinesia on the right. Gait was broad-based with some circumduction of the right leg and no freezing noted. The patient had exaggerated startle reaction that triggered spasms of the back and legs.

For several years, he was treated for presumed dystonia-parkinsonism although he had limited response to levodopa, trihexyphenidyl, baclofen, and Botox. Serologic workup on initial examination including ceruloplasmin, anti-gliadin antibodies, amino acid and organic acid analysis, thyroid stimulating hormone, HIV, zinc, and vitamins A and B1 was normal. Vitamin B12 was low at 84 pg/mL (reference range, 210-950 pg/mL) and was corrected with 1,000 μg intramuscular injections weekly in 2011 without any change in the progression of disease. In 2013, genetic testing included LRRK2 sequencing (normal), PARK2 sequencing (normal), PARK2 deletion/duplication (indeterminate result; single exon deletion), and PINK1 sequencing (indeterminate result), all performed at Athena Diagnostics.

At follow-up in 2021 when the patient was 57 years old, he was noted to have a markedly elevated anti-glutamic acid decarboxylase antibody (anti-GAD) of 494 nmol/L (reference, <0.02 nmol/L). The patient's neurologist initiated treatment with intravenous immune globulin, which is the first-line therapy for autoimmune encephalitis, including a broad array of anti-GAD–associated syndromes.

## RADIOGRAPHIC APPEARANCE

### Radiology Findings

Magnetic resonance imaging (MRI) of the brain in 2021 demonstrated diffuse volume loss in the right cerebellum, greater than expected for the patient's age. The volume loss was seen as expansion of the cerebrospinal fluid spaces in the posterior fossa around the right cerebellum, widening the cerebellar sulci with clear delineation of the folia. The right middle cerebellar peduncle was smaller than the left. The left cerebellar hemisphere had also developed focal atrophy superiorly. Importantly, no corresponding signal abnormality or contrast enhancement was seen. Additionally, the cerebellar findings were entirely disproportionate to the degree of cerebral atrophy. These findings developed between MRIs in 2011 and 2013, with more fulminant progression between MRIs from 2017 to 2021. The Figure shows initial MRIs from 2011 and corresponding MRIs from 2021.

**Figure.**
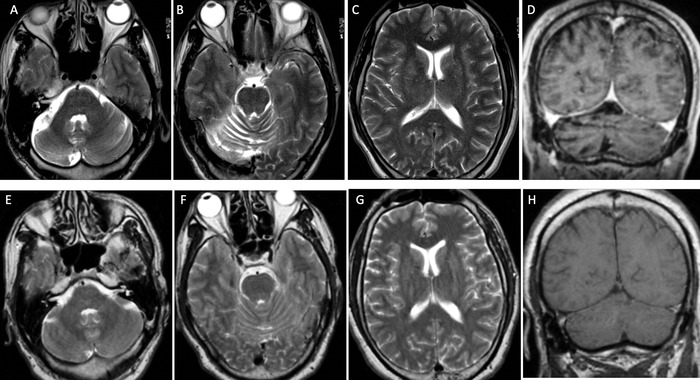
Magnetic resonance imaging of the brain from 2021 includes (A-C) axial T2 and (D) coronal contrast-enhanced T1 images showing diffuse volume loss throughout the right cerebellar hemisphere with expansion of cerebrospinal fluid spaces and widening of cerebellar sulci (A, B, D) that were greater than expected for the patient's age. (C) Supratentorial brain volumes are normal. Corresponding comparison images from 2011 (E-H) are normal with no cerebellar atrophy.

### Radiology Differential Diagnoses

Differential diagnoses for isolated cerebellar volume loss include developmental, ischemic, demyelinating, and inflammatory etiologies, as well as neurodegenerative disorders. Developmental lesions include the spectrum of cerebellar hypoplasia and malformations such as cerebellar dysplastic gangliocytoma. Cerebellar hypoplasia may be unilateral or global and related to genetic mutations or a prenatal insult.^2^ Cerebellar dysplastic gangliocytoma, or Lhermitte-Duclos disease, is a benign cerebellar malformative lesion of uncertain etiology. Imaging shows a striated corduroy appearance that typically enlarges one hemisphere and has associated signal changes.^3,4^ However, the right cerebellar volume loss in our patient was a progressive acquired lesion.

Ischemic lesions include cerebellar stroke and crossed cerebellar diaschisis. An infarct in the cerebellar hemisphere would produce focal encephalomalacia with increased T2 signal intensity conforming to a vascular distribution, but parenchymal signal was normal in our case. Crossed cerebellar diaschisis results from reduced blood flow in a cerebellar hemisphere contralateral to a supratentorial infarct, tumor, or other insult.^5^

Other insults to the cerebellum, including acute disseminated encephalomyelitis, JC virus granule cell neuronopathy, superficial siderosis, or cerebellitis, should also exhibit characteristic corresponding signal changes on different pulse sequences. Neurodegenerative diseases, including multisystem atrophy and hereditary cerebellar ataxias, were primary considerations for cerebellar volume loss. However, the patient had no other history to explain cerebellar volume loss, such as prior drug use (alcohol or phenytoin), chronic seizure disorder, or evidence of an underlying metabolic disorder, and the striking laboratory finding of markedly elevated anti-GAD antibodies ultimately cinched the diagnosis of chronic autoimmune encephalitis.

## DISCUSSION

The patient presented with a constellation of symptoms that suggested dystonia-parkinsonism. This syndrome may also present with gait difficulty and frequent falls. MRI showed progressive moderate atrophy of the right cerebellum and early involvement of the left superior cerebellum during a 10-year period. The laboratory analysis revealed very high levels of anti-GAD antibodies, confirming the diagnosis of autoimmune encephalitis.

While much is still being learned about this underrecognized disorder, numerous antibodies have been implicated, including anti-Hu, anti-Ma/Ta, anti-Yo, anti-Tr, anti-Ri, anti-CV2, and anti-GAD.^6^ Of these, anti-GAD antibodies have been specifically implicated with symptoms of exaggerated startle and rigidity.^6^ Symptoms vary depending on the patient and the antibody but may include memory loss, dementia, seizures, ataxia, dysarthria, and nystagmus.^1^ Patients with autoimmune encephalitis may also have psychologic features such as anxiety, depression, mood dysfunction, and hallucinations.^7^

Imaging findings for autoimmune encephalitis are variable and can range from none at all to a markedly abnormal examination with symptoms including cranial neuropathies, dystonia, chorea, psychosis, or seizures.^6^ Signal changes may affect the white matter, cortex, deep gray nuclei, and brain stem.^8^ Certain patterns have been identified, specifically cerebellar atrophy. Cerebellar atrophy is more commonly associated with paraneoplastic syndromes, although our patient had no known malignancy.

Perhaps the most well-known imaging presentation of autoimmune encephalitis is limbic encephalitis. Limbic encephalitis is inflammation of the limbic system, which includes structures such as the cingulate and parahippocampal gyri, hippocampus, and amygdala.^8^ The limbic system plays a major role in memory, olfaction, and emotion. Limbic encephalitis may produce increased T2/fluid-attenuated inversion recovery (FLAIR) signal and progressive atrophy in the limbic structures, particularly in the medial temporal lobe, sometimes mimicking herpes encephalitis.^8^

## CONCLUSION

This patient presented with symptoms of dystonia-parkinsonism and imaging findings of cerebellar atrophy. The correct diagnosis of autoimmune encephalitis was eventually made using serial brain imaging and serology positive for high levels of circulating anti-GAD antibodies.

## Notes

*This article meets the Accreditation Council for Graduate Medical Education and the American Board of Medical Specialties Maintenance of Certification competencies for Patient Care and Medical Knowledge.*
